# One-Year Monitoring of Prevalence and Diversity of Dairy Propionic Acid Bacteria in Raw Milk by Means of Culture-Dependent and Culture-Independent Methods

**DOI:** 10.3390/foods13121921

**Published:** 2024-06-18

**Authors:** Carola Bücher, Johanna Burtscher, Ulrike Zitz, Konrad J. Domig

**Affiliations:** 1 Austrian Competence Centre for Feed and Food Quality, Safety and Innovation (FFoQSI), Technopark 1D, 3430 Tulln, Austria; carola.buecher@ffoqsi.at; 2 Institute of Food Science, Department of Food Science and Technology, University of Natural Resources and Life Sciences, Vienna, Muthgasse 18, 1190 Vienna, Austria; ulrike.zitz@boku.ac.at (U.Z.); konrad.domig@boku.ac.at (K.J.D.)

**Keywords:** raw milk, propionic acid bacteria, qPCR, lithium glycerol agar, yeast extract lactate agar, cheese spoilage

## Abstract

Even low levels of dairy propionic acid bacteria (dPAB) can cause cheese defects, resulting in severe economic losses for the producers of selected raw milk cheeses. Therefore, routine quality control of raw cheese milk for dPAB contamination is essential if propionic acid fermentation is undesired. Although knowledge of dPAB contamination of raw milk is important to understand cheese spoilage, long-term dPAB screening data are outdated, and studies taking into account different farm management parameters and their potential influence on dPAB levels are scarce. This study aims to provide insight into the dPAB levels of raw milk over time, to identify farm management factors that potentially influence dPAB levels, and to compare a cultural yeast extract lactate agar (YELA) and lithium glycerol agar (LGA) and a culture-independent method (qPCR) for dPAB quantification with respect to their applicability in routine quality control for the dairy industry. For this purpose, bulk tank milk from 25 dairy farms was screened for dPAB contamination over a one-year period. We were able to identify significant differences in the dPAB contamination levels in raw milk depending on selected farm-specific factors and observed relationships between the different types of milking systems and dPAB contamination levels in raw milk. When dPAB were quantified by cultivation on YELA, strong overgrowth of commensal microbiota impeded counting. Therefore, we conclude that quantification on LGA or by qPCR is preferable. Both methods, colony counting on LGA as well as quantification of dPAB using qPCR, have advantages for the application in (routine) quality control of raw milk, one being low-tech and inexpensive, the other being fast and highly specific, but the detection of (low level) dPAB contamination in raw milk remains a challenge.

## 1. Introduction

The group of propionic acid bacteria (PAB) underwent a taxonomic reclassification in 2016 [1]. Today, the term dairy propionic acid bacteria (dPAB) is used to describe the four species: *Propionibacterium freudenreichii*, *Acidipropionibacterium acidipropionici*, *A. jensenii*, and *A. thoenii* [1]. Even though *P. freudenreichii* is used as a starter culture in the production of generic and raw milk Emmental cheese, wild type dPAB are feared due to their potential to enhance propionic acid fermentation in raw milk Emmental cheese (protected designation of origin, PDO) or to cause undesired propionic acid fermentation in other raw milk cheeses such as Vorarlberger Bergkäse (PDO), Sbrinz (PDO) or Parmigiano Reggiano (PDO) [2,3,4]. If dPAB are present in the cheese milk used to make these cheeses, they can cause unwanted eyes, reddish-brown spotting, and cracks in the cheese matrix [2]. They also affect the sensory characteristics of the cheese and may lead to poor storage quality [2,5]. For these cheeses, it is therefore recommended to use raw milk containing less than 10–30 dPAB/mL raw milk to avoid undesired propionic acid fermentation, and regular quality controls of the raw cheese milk are essential [2].

The quantification of dPAB in raw milk by classical cultivation is frequently performed for routine quality controls of raw milk intended for cheese production, and there are different culture media available [6]. Amongst these different culture media, yeast extract lactate medium and lithium glycerol medium are media that are mentioned specifically and regularly in the context of dPAB enumeration from milk and dairy products [7,8,9]. One of the greatest drawbacks when cultivating dPAB is their slow growth, which requires an incubation period of seven to ten days, and the need for an identification step after quantification due to the growth of accompanying raw milk microbiota [7,10]. However, some authors have reported even longer incubation times of up to one month to ensure the inclusion of slow-growing dPAB strains [11]. In order to circumvent these long incubation times and the additional identification step, a quantification by qPCR is possible. Turgay et al. [3,12] have developed a multiplex qPCR assay targeting *P. freudenreichii* and three dairy-relevant *Acidipropionibacterium* species, which has the advantage of a greatly reduced workload compared to previously developed assays that targeted each species separately. However, detecting low dPAB levels that can already cause cheese defects can be a challenge by qPCR, especially as DNA extraction from raw milk can be a demanding task due to the high fat and protein content of the matrix [13].

Focusing on the more recent literature, there is limited information available on the dPAB contamination levels in raw milk, and to the best of our knowledge, none have monitored the same farms over an extended time period of approximately one year [7]. The studies that have focused on this issue were published more than twenty years ago. On the one hand, this is problematic in terms of the taxonomic reclassification, which is discussed in more detail in Bücher et al. [6], and on the other hand, there have been many changes in farm management, also considering the increasing number of farms that transition to automatic milking systems (AMS; [14,15]). Therefore, our focus was to gain insights into the variation of dPAB levels in raw milk from 25 Austrian dairy farms over a period of one year and to learn more about the abundance of the four dPAB species in different types of raw milk, such as conventional, organic, or hay milk. We also wanted to determine if we could identify significant differences in dPAB contamination levels in raw milk depending on factors such as farm size, type of milking system, or environmental conditions (e.g., season, temperature, rainfall). To this end, we analyzed a total of 426 raw milk samples, of which 88 samples were used for pre-screening to compare the performance of the above-mentioned culture media and to select the farms for long-term screening. The remaining 338 raw milk samples were analyzed with the best-performing culture medium and by qPCR to compare the suitability of culture-dependent and culture-independent approaches for quantifying dPAB contamination in raw milk, with a particular focus on their application in routine quality control in the dairy industry.

## 2. Materials and Methods

### 2.1. Farm Selection and Pre-Screening

**Figure 1:**
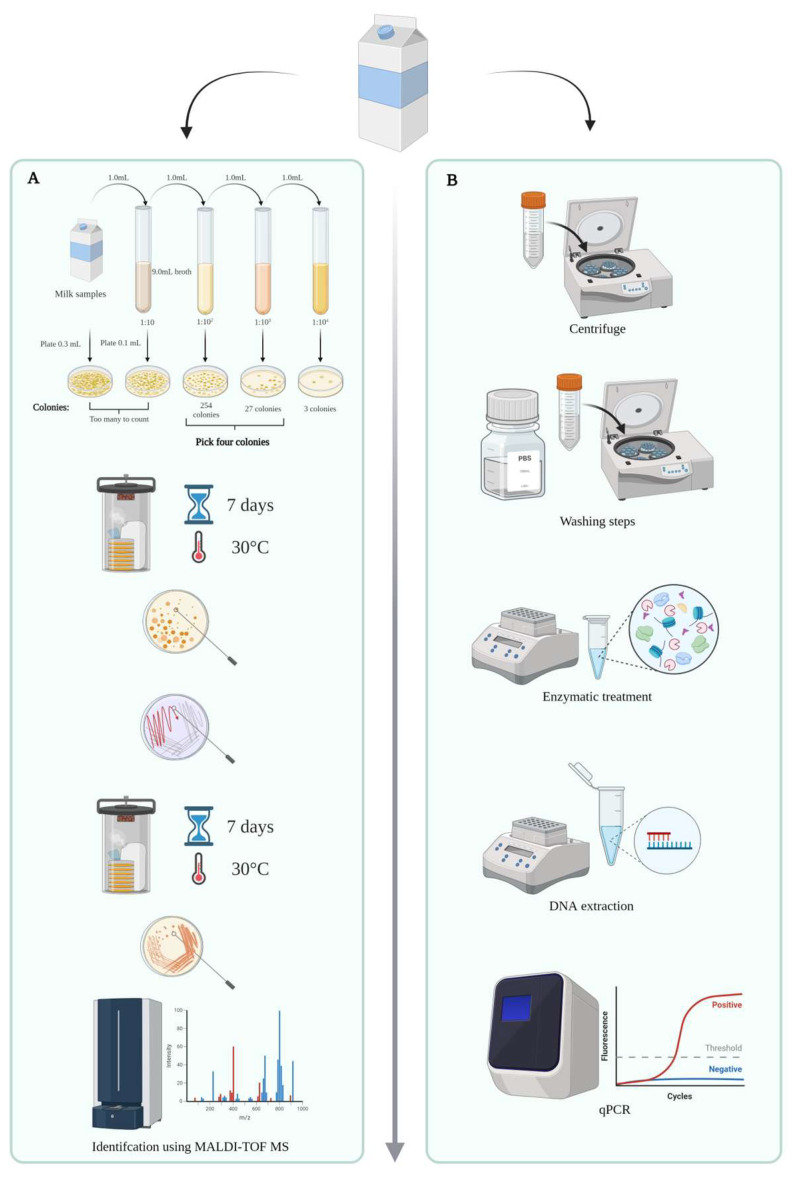
Overview of the sample analysis scheme for the quantification of dPAB in raw milk using a culture-dependent approach indicated by the letter (**A**) and a culture-independent approach indicated by the letter (**B**). Created with BioRender.com.

A total of 45 dairy farms, all supplying the same cheese dairy, were selected by the dairy to participate in pre-screening. The 45 farms produced different types of milk: conventional milk (22/45 farms), hay milk (11/45 farms), organic milk (8/45 farms), and organic hay milk (4/45 farms). Hay milk, also sometimes called pasture milk, is a traditional specialty protected by the European Union’s Traditional Specialty Guaranteed (TSG) label that has recently been appointed a global agricultural heritage by the Food and Agriculture Organization of the United Nations [16,17,18]. Hay milk-producing farms are prohibited from feeding silage, and cattle must be kept in loose barns or pasture grazing for at least 120 days per year [16,17]. Milk samples for the pre-screening were obtained during the second and last week of May 2022. The samples were automatically collected by the tanker at the farm during the milk collection and frozen by the dairy after milk delivery. A total of 88 raw milk samples were shipped in coolers to our laboratory, where they were kept frozen at −30 °C until analysis. An overview of the sample analysis scheme is given in Figure 1, with the culture-dependent analysis scheme indicated by the letter A. For analysis, the milk samples were thawed and homogenized by inversion 25 times according to ISO 6887-5:2010, and serial dilutions were created in maximum recovery diluent (MRD, LabRobot Products AB, Stenungsund, Sweden) [19]. For the pre-screening, two different media were used to quantify dPAB: yeast extract sodium lactate agar (YELA; 10 g peptone, 20 mL sodium lactate, 10 g yeast extract, 328 mg K_2_HPO_4_, 56 mg MnSO_4_, 15 g bacteriological agar; [20]) and lithium glycerol agar (LGA; 10 g lithium lactate, 10 g peptone, 6 g glycerol, 1 g powdered milk, 50 mg bromocresol purple, 328 mg K_2_HPO_4_, 56 mg MnSO_4_, 12 g bacteriological agar; [9]). Analyses were performed in duplicate. Both media were inoculated with 1 mL of homogenized raw milk or 0.1 mL of diluent using the spread plate method and incubated anaerobically (80% N_2_, 10% CO_2_, 10% H_2_) either using the Whitley jar gassing system (Don Whitley Scientific, Bingley, UK) or in an anaerobic chamber at 30 °C for seven days. After seven days, presumptive dPAB colonies were quantified, and four colonies per sample were subcultured on the corresponding medium and grown colonies were subsequently identified by matrix-assisted laser desorption/ionization time-of-flight mass spectrometry (MALDI-TOF MS) by employing the Bruker Biotyper System. If the identification by MALDI-TOF MS remained inconclusive (MALDI-TOF MS Score < 1.7), 16S rRNA gene sequencing was performed. Four presumptive dPAB colonies were selected, and the identification was performed as described in Bücher et al. [4]: of each streak, biological material was transferred on an MSP 96 polished steel target plate, creating a thin film, which was then treated according to the “extended direct transfer method”. First, it was overlaid with 1 μL of 70% formic acid (Carl Roth, Karlsruhe, Germany), which was dried at room temperature, and 1 μL of a-cyano-4-hydroxycinnamic acid (HCCA; Bruker Daltonics, Bremen, Germany) matrix solution was added. Next, measurements were performed using a Microflex LT mass spectrometer (Bruker Daltonics, Bremen, Germany; method “MTB_AutoX”). The generated spectra were identified using the Bruker MTB database (Version 9, Revision K, 2022), the BIOTECON Diagnostics D-Mass-02 database (Version 1), and an in-house database with additional dPAB spectra. When identification was performed by 16S rRNA gene sequencing, DNA extraction was performed using Chelex 100, as described by Walsh et al. [21]. 16S rRNA gene amplification, subsequent PCR product purification, and sequence analysis were performed according to Brändle et al. [22], but using AccuStart II PCR ToughMix (Quantabio, Beverly, MA, USA) and sequencing at Microsynth (Vienna, Austria). If the sequence analysis yielded results with identities ≥ 98%, the results were considered reliable. Based on the results of the pre-screening, the farms were divided into three groups (“dPAB low”, “dPAB medium”, and “dPAB high”) according to the level of dPAB contamination. Within the “dPAB low” group, dPAB levels were ≤2.0 log cfu dPAB/mL raw milk; the “dPAB medium” group included farms with dPAB levels in the range of 2.0–2.4 log cfu dPAB/mL raw milk and the “dPAB high” group included farms with dPAB levels ≥ 2.4 log cfu dPAB/mL raw milk.

### 2.2. Farm Data and Sampling Information

Of the 45 farms from the pre-screening, 25 were selected for regular screening: Eight farms from the “dPAB low” and eight from the “dPAB medium” group, and nine farms from the “dPAB high” group. When selecting farms for long-term monitoring, we aimed to achieve a balance so that all milk types, milking systems, and farm sizes were equally represented. Of these 25 farms, 11 farms produce conventional milk, six farms each produce hay and organic milk, and two farms produce organic hay milk. Sampling started in mid-September 2022, and samples were sent every two to three weeks until January 2023. From January to March 2023, samples were sent once a month because less variation in dPAB levels was expected due to winter temperatures that made grazing impossible, so the cattle were kept indoors. At the end of March, we returned to the original sampling frequency, and sampling continued until August 2023. In total, we received milk samples from 15 collection dates, but samples from one collection date arrived spoiled due to a shipping error and were not analyzed. Because we needed approximately 25 mL of milk, two milk samples drawn by the sampler on the milk tanker at consecutive milk collections were pooled.

Weather data were recorded for each sampling date (morning temperature, daytime temperature, rainfall/dry). In addition, the transition from pasture to barn and back was recorded. We obtained information on the amount of milk (kg) per year produced by each farm, which ranged from 28,914 kg/year to 517,142 kg/year. In total, three farms used a milking robot, and three farms transitioned from a milking parlor to a milking robot during our sampling period. Ten farms used a bucket or a pipe milking system, and the remaining nine farms had a milking parlor. We grouped farms with a bucket milking system or a pipe milking system together, and they are referred to as farms using a pipe milking system. We were provided with the colony counts (CC) from the routine analysis commissioned by the dairy. Because we could only be supplied with milk samples if the dairy did not use the milk tanker samples for routine analysis, the CC analyses were performed on different milk samples. We calculated the mean CC using the counts from 10 days before and 10 days after our sampling date.

### 2.3. Milk Screening Using Microbiological Methods

For microbiological quantification of dPAB counts, the samples were treated as described above but with a slight change in protocol: quantification was only performed using LGA, but not YELA, followed by an identification step using MALDI-TOF MS and 16S rRNA gene sequencing, if the identification by MALDI-TOF MS remained inconclusive (MALDI-TOF MS Score < 1.7). In addition, 20 mL of each homogenized milk sample was transferred into a 50 mL centrifuge tube and set aside for DNA extraction.

### 2.4. Milk Screening Using a Culture-Independent Approach

Along with the dPAB quantification by microbiological methods, we used a multiplex qPCR assay previously published by Turgay et al. [3,12]. One primer system targets *P. freudenreichii*, while the other primer system combines the quantification of the three dairy-relevant thermosensitive species: *A. acidipropionici*, *A. jensenii,* and *A. thoenii* (3dPAB). An overview of the sample analysis scheme is given in Figure 1, and the culture-independent approach is indicated by the letter B.

### 2.5. DNA Extraction

20 mL of raw milk was centrifuged (Eppendorf Centrifuge 5920R, Eppendorf SE, Hamburg, Germany) at 4 °C for 20 min at 8000 rpm. Next, the milk fat was removed using a sterile swab (Heinz Herenz Medizinalbedarf GmbH, Hamburg, Germany), and the milk was discarded. The remaining pellet was homogenized in 20 mL sterile phosphate-buffered saline (PBS, Merck KGaA, Darmstadt, Germany), centrifuged, and the remaining milk fat was removed again. This washing step with PBS was repeated twice. Next, following the protocol of Doyle et al. [23], 120 µL lysozyme (50 mg/mL, Merck KGaA, Darmstadt, Germany) and 67 µL mutanolysin (50 U/mL, Merck KGaA, Darmstadt, Germany) were added to the pellet and gently mixed by pipetting. This mixture was transferred to a 1.5 mL reaction tube that was placed in a ThermoMixer C (Eppendorf SE, Hamburg, Germany) and heated to 55 °C for 15 min while shaking at 300 rpm. After heating, 37 µL proteinase K solution (20 mg/mL, Merck KGaA, Darmstadt, Germany) was added, and the heating step was repeated. Afterwards, the mixture was centrifuged for 5 min at room temperature at 14,000× *g,* and the supernatant was removed. The remaining pellet was used for DNA extraction with 200 µL Chelex 100 resin (Bio-Rad Laboratories Ges.m.b.H., Vienna, Austria) as described in Bücher et al. [4].

### 2.6. Creation of a Standard Curve

In order to create a combined *P. freudenreichii*—3dPAB standard, the type strains of the four dPAB species (*P. freudenreichii* DSM 20271; *A. acidipropionici* DSM 4900; *A. jensenii* DSM 20535; *A. thoenii* DSM 20275) were grown on LGA under the conditions described above. Next, overnight cultures were grown in lithium glycerol broth: 30 mL of *P. freudenreichii* overnight culture and 10 mL each of *A. acidipropionici*, *A. jensenii,* and *A. thoenii* overnight culture were added to 170 mL UHT milk (3.5% fat content, Milfina) to obtain a final concentration of 8.3 log cfu *P. freudenreichii* and 8.1 log cfu 3dPAB. Five replicates of 20 mL of each spiked sample (*P. freudenreichii* and 3dPAB) were transferred into a 50 mL centrifuge tube (Greiner Bio-One, Frickenhausen, Germany), which was used for DNA extraction as described above. Then, each 10 µL of the *P. freudenreichii* and 3dPAB DNA extracts were mixed in a 1.5 mL reaction tube, 80 µL of ultrapure water (Merck KGaA, Darmstadt, Germany) was added, and the combined *P. freudenreichii*–3dPAB standard was serially diluted up to 10^−7^. These were used for qPCR measurements as described below. The standard curve was created using the Magnetic Induction Cycler PCR Software (Version 2.12.7, Bio Molecular Systems, Upper Coomera, Australia). From the remaining spiked milk samples, serial dilutions were created as described above, and *P. freudenreichii* and 3dPAB counts were quantified on LGA, with a subsequent identification step to ensure that all species were represented.

### 2.7. Real-Time Quantitative PCR

A total of 5 µL template DNA was used per qPCR reaction with a total volume of 20 µL, which also contained 10 µL Luna Universal Probe qPCR Mastermix 2x (New England Biolabs, Frankfurt, Germany), 0.8 µL of a primer mix containing all four primers (10 µM; Microsynth AG, Balgach, Switzerland), 0.4 µL probe mix containing both probes (10 µM; Microsynth AG), and 3.8 µL of ultrapure water. For each run, ultrapure water was used as a no-template control, DNA from the strains *P. freudenreichii* DSM 4902 and *A. acidipropionici* LMG 16449 (each alone and a 1:1 mixture) were used as positive controls, and *Enterococcus faecalis* DNA from a cheese isolate previously identified by 16S rRNA gene sequencing was used as a negative control. Additionally, two concentrations of the *P. freudenreichii*—3dPAB standard were included in each run. PCR reactions were carried out in a MIC Magnetic Induction Cycler (Bio Molecular Systems, Upper Coomera, Australia) with the following qPCR conditions: an initial denaturation step at 95 °C for 60 s, followed by 45 cycles at 95 °C for 15 s and 60 °C for 30 s, and a final hold at 60 °C for 30 s. All samples were measured in duplicate.

### 2.8. Statistical Analysis

Statistical analysis was performed using JMP Pro Software (JMP Pro, 17.0.0, 64-bit version, 2022 JMP Statistical Discovery LLC, Cary, NC, USA) and results were considered significant if *p* < 0.05. To compare dPAB levels quantified on LGA and by qPCR, *P. freudenreichii* and 3dPAB levels determined by qPCR were summed up prior analysis, as non-pigmented 3dPAB cannot be distinguished from *P. freudenreichii* on LGA. The results of the dPAB quantification and the CC determination were log_10_ transformed prior to analysis. *P. freudenreichii* and 3dPAB levels below the limit of quantification (*LOQ*) determined by qPCR, as well as samples in which no amplification could be detected, were included in the statistical analysis; the latter with an approximation of 0 log, as all these results provide important information for model generation. To identify process variables potentially associated with dPAB contamination in raw milk, a partition decision tree, as described by Burtscher et al. [24], was used. The decision tree was validated on 30% of the dataset, and variables were identified based on the results. Mixed models for repeated measures with AR(1) covariance structure were used to investigate the influences of the process variables on the results of dPAB quantification (qPCR/LGA) over the one-year sampling period. The milking system, milk yield (corresponding to the farm size), season, and milk type served as fixed effects and the suppliers as repeated effects covariance parameter (subjects), with sampling taking place repeatedly in defined weeks. Multiple comparisons (Tukey’s HSD method) were used to identify significant differences in dPAB contamination levels of the above-named process variables. Spearman’s rank correlation coefficient was used to test the data for potential correlations between the dPAB levels quantified on LGA and by qPCR and CC, as well as the *P. freudenreichii* and 3dPAB levels quantified by qPCR. Passing Bablok regression was used to assess the differences between the cultural LGA and the non-cultural qPCR quantification approaches.

## 3. Results and Discussion

### 3.1. Comparison of the Culture Media YELA and LGA

**Figure 2:**
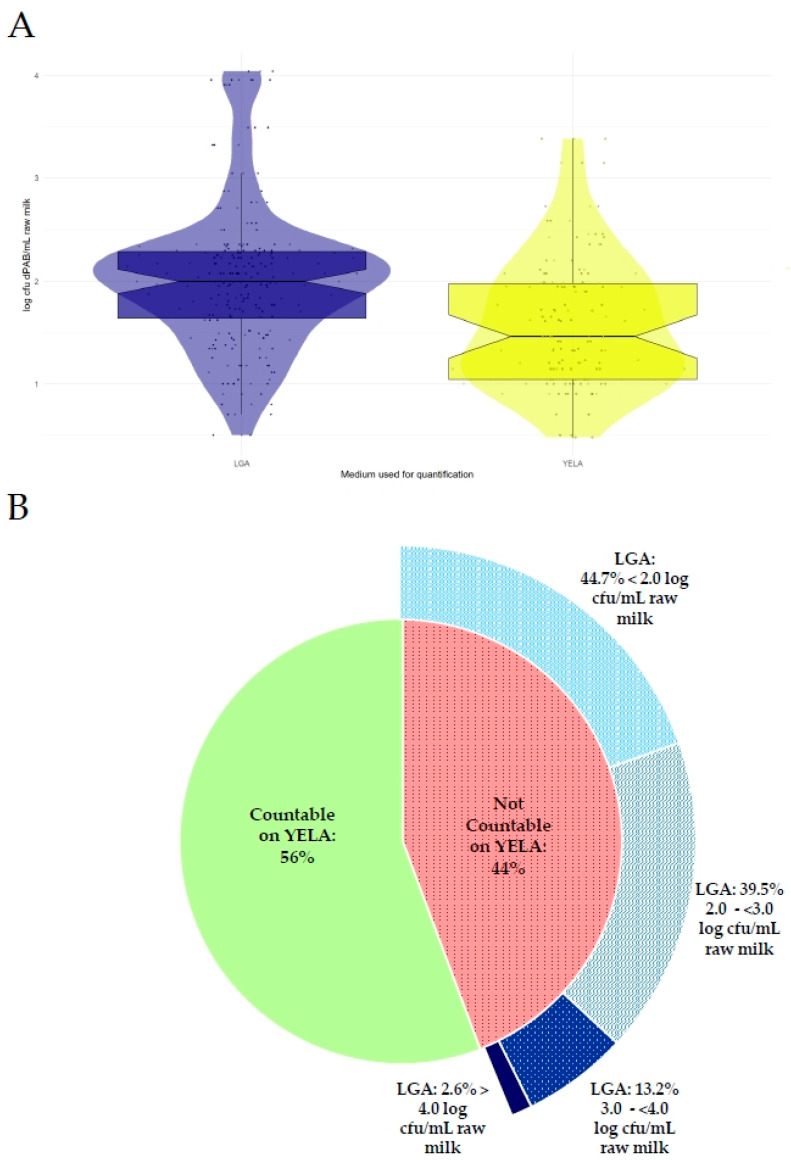
(**A**) Box plots showing the dPAB levels in raw milk according to the medium used for quantification. dPAB levels quantified on lithium glycerol agar are shown in blue, and dPAB levels quantified on yeast extract lactate agar (YELA) are shown in yellow. Box plots represent the median, the first, and the third quartile (25%/75% of the data), and the whiskers extend to the lowest and highest values that are not outliers (max. 1.58 × interquartile range). Each dot represents one sample; dots beyond the whiskers are outliers. (**B**) shows the percentage of samples that could be counted on YELA and the corresponding dPAB levels quantified on LGA for the 44% of samples that could not be counted on YELA.

The quantified dPAB levels in the 88 samples from the pre-screening were within similar ranges on both growth media, ranging from <1.0 log cfu dPAB/mL raw milk to 3.4 log cfu dPAB/mL raw milk when quantified on YELA and from <1.0 log cfu dPAB/mL to 4.0 log cfu dPAB/mL when quantified on LGA. However, we observed differences in the performance of the two-culture media. The mean dPAB contamination level quantified on YELA was 0.4 log cfu dPAB/mL lower than on LGA, as shown in Figure 2A. While counting dPAB colonies was possible for all but one sample on LGA, only 56% of the samples could be counted on YELA. Counting the remaining 44% of the samples was not possible on YELA due to overgrowth by non-target microbiota. However, the analysis of these samples using LGA showed that relevant levels of dPAB were present in raw milk, as shown in Figure 2B: 44.7% of the samples contained dPAB at levels < log 2.0 cfu dPAB/mL raw milk, 39.5% contained between log 2.0–<3.0 cfu dPAB/mL, 13.2% between log 3.0–<4.0 cfu dPAB/mL raw milk, and 2.6% > log 4.0 cfu dPAB/mL raw milk. As YELA is widely used for the quantification of dPAB in routine quality control, these results indicate a problematic underestimation of dPAB contamination, considering that dPAB levels >1.5 log cfu dPAB/mL, which can be detrimental to raw milk cheese production if propionic acid fermentation is undesirable, may have been missed.

As LGA and YELA are both elective media, an additional identification step is necessary. In total, 123 colonies were selected from LGA and streaked for identification, but only 35 colonies were picked from YELA for subsequent identification by MALDI-TOF MS. The difference in the number of isolates is due to the high number of samples that could not be quantified on YELA due to non-target overgrowth. On YELA, 57.1% of the isolates (*n* = 35) were identified as *P. freudenreichii*, in addition to *Lacticaseibacillus* spp. (14.3%), *Lactiplantibacillus plantarum* (8.6%), *Enterococcus faecium* (8.6%), *Pediococcus pentosaceus* (5.7%), *Acidipropionibacterium jensenii* (2.9%) and *Staphylococcus borealis* (2.9%). In total, only 60.0% of the YELA isolates belonged to the dPAB group; the remaining 40.0% were mainly lactic acid bacteria. In comparison, 99.2% of all isolates identified from LGA (*n* = 123) belonged to the dPAB group. The relative abundance of *P. freudenreichii* on LGA was 87.8%, followed by *A. thoenii* (6.5%), *A. jensenii* (3.3%), *A. acidipropionici* (1.6%), and *Staphylococcus* spp. (0.8%). LGA showed higher selectivity for dPAB, but the greater abundance of identified dPAB on LGA also demonstrates that dPAB are more easily distinguished from the accompanying microbiota when grown on LGA. Considering the previous literature, the greater selectivity of LGA for the enumeration of dPAB in raw milk compared to YELA has already been demonstrated [7,8]. Nevertheless, YELA is still widely used in routine analysis of raw milk in milk quality laboratories.

Attempts have been made to improve the media performance of YELA by adding 2,3,5-Triphenyltetrazolium chloride (TTC), which causes a red coloration of grown dPAB colonies. However, the addition of TTC to YELA did not yield satisfactory results, as TTC appeared to inhibit the growth of some of the dPAB strains tested [8].

As 44% of the samples could not be counted on YELA due to a lack of selectivity resulting in an overgrowth by non-target microbiota, we decided to exclude this medium from further analysis.

### 3.2. Comparison of a Cultural and a Culture-Independent Approach for the Long-Term Monitoring of dPAB in Raw Milk

Based on the contamination levels quantified in the raw milk samples during the pre-screening, we were able to select 25 farms for the long-term raw milk screening using LGA and a multiplex qPCR assay for the quantification of dPAB. The dPAB levels in the 338 raw milk samples analyzed by qPCR and on LGA ranged between no dPAB in 1 mL raw milk (LGA, qPCR) to 4.2 log cfu dPAB/mL raw milk quantified on LGA and 5.1 log cfu dPAB/mL raw milk quantified by qPCR. In total, 4.4% (15/338) of the samples quantified on LGA were presumably free of dPAB contamination, while 20.7% (70/338) of the samples quantified by qPCR were presumed to be free of dPAB. Only in 3/338 samples was dPAB contamination detected in the raw milk samples by qPCR, but not on LGA. However, the dPAB levels quantified by qPCR were also very low; two samples contained levels below the *LOQ* (<1.7 (*P. freudenreichii*)/1.5 (3dPAB) log cfu/mL raw milk), the third sample contained 1.7 log cfu dPAB/mL raw milk. Of all 70 samples that were presumed to be free of dPAB based on the qPCR measurements, the results of 12 samples were in good agreement when cultivated on LGA. However, this was not the case for all remaining samples. While the contamination levels of 26 samples quantified on LGA were low (<1.0 log cfu dPAB/mL raw milk), 25 samples had contamination levels between 1.0–<2.0 log cfu dPAB/mL raw milk on LGA. Six samples that were presumably dPAB-free based on the qPCR results had high contamination levels between 2.0–<3.0 log cfu dPAB/mL when quantified on LGA, and the contamination level of one presumably dPAB-free sample even exceeded 3.0 log cfu dPAB/mL raw milk (LGA). Indeed, 45.7% of these supposedly dPAB-free 70 samples exceeded the critical limit of 30 dPAB/mL raw milk, which is recommended to stay below in order to avoid propionic acid fermentation in raw milk cheeses [2]. The fact that several samples containing dPAB above the critical dPAB level on LGA were undetected by qPCR is a drawback of the qPCR assay. Of the 263 samples that tested positive for dPAB contamination by qPCR, 254 samples (96.5%) were contaminated with *P. freudenreichii*, 191 samples (72.6%) were contaminated with 3dPAB, and (68.4%) both *P. freudenreichii* and 3dPAB were identified in 180 samples. Across all samples, the contamination levels ranged from no *P. freudenreichii*/3dPAB in 1 mL raw milk, up to 5.1 log cfu *P. freudenreichii*/mL raw milk and 3.0 log cfu 3dPAB/mL raw milk, respectively. Because we used the 3dPAB primer system, further discrimination of the *Acidipropionibacterium* species was not possible. However, considering that the 3dPAB primer system leads to a significant reduction in workload and that the three acidipropionibacteria often do not survive the cheesemaking process because they are less thermotolerant, the inability to discriminate between the species is less of a concern.

**Figure 3:**
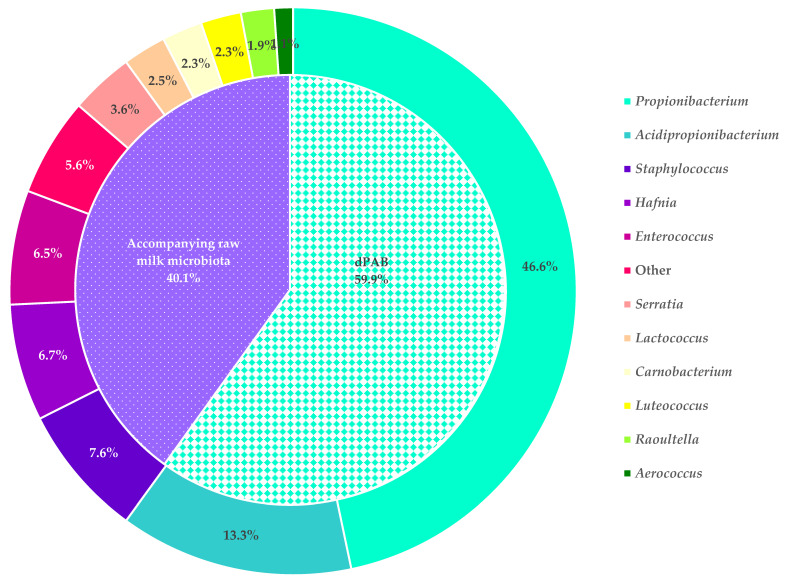
Distribution of *Propionibacterium* spp., *Acidipropionibacterium* spp. And genera from the accompanying raw milk microbiota (depicted clockwise) among the raw milk isolates identified by MALDI-TOF MS, given in percent of the total number of isolates ident identified. “Other” includes the following genera that represented less than one percent of the total number of isolates: *Alcaligenes*, *Bacillus*, *Brachybacterium*, *Buttiauxella*, *Citrobacter*, *Clostridium*, *Corynebacterium*, *Enterobacter*, *Erwinia*, *Escherichia*, *Ewingella*, *Globicatella*, *Klebsiella*, *Kluyvera*, *Kosakonia*, *Lelliottia*, *Leuconostoc*, *Levilactobacillus*, *Limosilactobacillus*, *Paraclostridium*, *Pediococcus*, *Pseudescherichia*, *Rahnella*, *Streptococcus*, *Vagococcus* and *Yersinia*.

Although more selective than YELA, LGA is still a non-selective medium and a subsequent identification step is required to confirm the presence of dPAB in the sample. This was done by MALDI-TOF MS. We chose three colonies with typical dPAB morphology and one atypical colony from each sample for identification. On average, we identified *P. freudenreichii* in 86.7% of the raw milk samples, *A. jensenii* in 26.6% of the milk samples, *A. thoenii* in 12.1% of the milk samples, and *A. acidipropionici* in 1.2% of the samples. In total, 58.9% of the samples were contaminated with one dPAB species, 29.6% of the samples were contaminated with two dPAB species, and 3.0% of the samples were contaminated with three dPAB species. None of the samples contained all four dPAB species. Overall, 4.4% of the samples were presumed to be free of dPAB when quantified by LGA, but subsequent identification by MALDI-TOF MS did not yield the same results. In more samples (8.6%), no dPAB were identified by MALDI-TOF MS, indicating that 4.2% of the samples on LGA were false positive. This highlights one of the major weaknesses of dPAB enumeration on LGA: the colony count relies entirely on a distinct morphology that dPAB exhibit on LGA, combined with a color change of the medium from purple to yellow due to the change in pH. The growth of salt-resistant cocci, presumably enterococci, on LGA, has been reported previously by Thierry and Madec [7], and depending on the colony size, they can be mistaken for dPAB. We found that species such as *Luteococcus japonicus* or *Carnobacterium maltaromaticum* also closely resemble dPAB on LGA and even small *Staphylococcus* spp. colonies can be difficult to distinguish, making the counting of dPAB difficult. In the worst case, dPAB levels in milk can be greatly over- or underestimated when quantified on LGA, as the results of dPAB enumeration are highly dependent on the operators’ experience and ability to distinguish dPAB from the accompanying raw milk microbiota. Excluding the measurements that led to an inconclusive identification by MALDI-TOF MS (MALDI-TOF MS Score < 1.7), we identified 1218 isolates. In addition, 105 isolates were identified by 16S rRNA gene sequencing. Because we considered the results of all isolates with a MALDI-TOF MS score ≥ 1.7, identification data are provided at the genus level. This is illustrated in Figure 3, which shows the proportions of dPAB and the identified genera of accompanying raw milk microbiota that we detected in the raw milk samples. Of the 1218 isolates, 59.9% belonged to the dPAB group, and the other isolates belonged to the accompanying raw milk microbiota. Among the genera identified from the raw milk were *Staphylococcus* (7.6%), *Hafnia* (6.7%), and *Enterococcus* (6.5%). We also identified *Serratia*, *Lactococcus*, *Carnobacterium*, *Luteococcus*, *Raoultella,* and *Aerococcus*, but each of these six genera accounted for less than 5% of the total number of isolates. The presence of these genera in raw milk was not surprising, as these have been previously associated with raw milk, the stable environment, or as potential agents causing intramammary infections [4,25,26,27]. Genera representing less than one percent of the total isolates were grouped as “other”. All in all, these results underscore the problem of the lack of specificity when cultivating dPAB, which has already been addressed.

A potential lack of specificity is not a problem when the samples are analyzed by qPCR, provided that the assay has been properly established beforehand. However, there are other factors, such as DNA extraction or various pipetting steps, which may complicate dPAB quantification by qPCR in raw milk samples. These issues have already been discussed in the literature but are still relevant [13,28]. In addition to all those technical challenges, very low dPAB levels of 10–30 cfu/mL in raw milk are already sufficient to cause cheese defects. Thus, the detection of dPAB is essential. Levels as low as 10 cfu dPAB/mL have been previously detected by qPCR, but this can be challenging, and quantification of such low levels was not possible [3,28]. Turgay et al. [12] used a laborious pre-treatment of the milk samples prior to DNA extraction to prevent the loss of dPAB cells with the milk fat, and while the results were convincing, the suitability for routine quality control of raw milk samples is questionable. We chose to extract DNA from a larger volume of raw milk (20 mL), five times the volume used by Turgay et al. [3], to ensure the detection of low-level dPAB contamination. DNA extraction kits often provide better-quality DNA extracts, but they are usually used with only one mL of sample material. In preliminary tests, we observed higher DNA yields when using Chelex 100 than when using DNA extraction kits. In view of potential use in routine quality control, Chelex 100 extraction also requires fewer steps and is less expensive than DNA extraction kits. Therefore, we used Chelex 100 extraction, although the disadvantage of this method is the comparatively impure DNA extracts [28,29]. The washing steps, sample treatment, and DNA extraction using Chelex 100, in combination with the PCR assay published by Turgay et al. [3], resulted in a *LOQ* of 30 cfu 3dPAB/mL raw milk and 50 cfu *P. freudenreichii*/mL raw milk, which is comparable to the *LOQ* reported by Turgay et al. [3,12]. This is based on the definition in Ricchi et al. [30], meaning that all replicates had to produce a positive result, and the coefficient of variation is less than 25%. We have also adopted their definition of the limit of detection (*LOD*), which is represented by a 95% positive call [30,31]. The *LOD* in our study was lower than the *LOQ* to the power of ten. The primer efficiency of the primer systems (*P. freudenreichii*/3dPAB) was 1.0 and 0.85, respectively, and an *R*^2^ of 0.99 and 0.98 (*P. freudenreichii*/3dPAB) was obtained. Although the *LOQ* is sufficient to analyze raw milk samples within the “dPAB low” category, it is still not sufficient to identify all samples that exceed the cheese-dependent threshold level of 10–30 cfu dPAB/mL raw milk. Furthermore, the share of presumably dPAB-free samples was much higher when quantified by qPCR. This could be due to less pure DNA extracts caused by the Chelex 100 extraction or to the small sample volume that is transferred into the qPCR reaction: although we used 5 µL of Chelex 100 extract for each qPCR reaction, this represents only 2.5% of the extracted volume. In addition, we cannot exclude the loss of dPAB during the washing steps, as they are potentially bound to milk fat, resulting in a higher percentage of negative qPCR results compared to quantification on LGA. All in all, this underlines the well-documented challenges of DNA extraction and qPCR detection of (low-level) bacterial contamination in raw milk.

Method harmonization and comparability are essential when a method is used in routine quality control. Therefore, we investigated potential correlations between the quantification of dPAB cultured on LGA and quantified by qPCR. Because there is little information available, and most studies are limited to *P. freudenreichii*, we also wanted to know if the presence of *P. freudenreichii* and the 3dPAB species determined by qPCR in the raw milk samples correlated. As older literature mainly employed cultural techniques for dPAB detection, the quantification and differentiation of *P. freudenreichii* and 3dPAB was impracticable. We observed a moderate, positive correlation between the *P. freudenreichii* and 3dPAB levels (*p* < 0.0001, *r* = 0.58) quantified by qPCR and a strong, positive correlation between quantification results on LGA and by qPCR (*p* < 0.0001, *r* = 0.68). We also wanted to determine the degree of agreement between the two quantification methods. This was done by Passing Bablok regression analysis, and we identified proportional systematic effects: the quantification by qPCR resulted in approximately 1.7 times higher detected dPAB levels than the analysis of the same sample on LGA. The higher the dPAB levels in the sample analyzed, the stronger the effect. This is probably due to the detection of DNA from dead bacterial cells or dPAB entering a viable but non-culturable (VBNC) state that is detected by qPCR but not on LGA [6]. Using the qPCR assay as we did, it is also impossible to distinguish between living and dead bacterial cells. Depending on the proportion of live/dead dPAB in raw milk, this differentiation may be critical for the potential use of the method for routine analysis in the dairy industry. However, little is known about the extent of dead or VBNC dPAB cells in raw milk, and results are likely to be strongly influenced by farm management [4]. The use of propidium monoazide (PMA) would be a possible solution to allow live/dead differentiation, which has been successfully used in the past to quantify bacterial loads in food samples, including raw milk [32,33]. However, this would double the workload as the assay would have to be performed with and without PMA, making it a laborious process, hence being a drawback for its use in routine quality control. All in all, the differences in the quantification of dPAB by PCR approaches and cultural methods are not surprising and have been reported before for the quantification of other microorganisms [30,34]. Ricchi et al. [30] compared the quantification by different PCR approaches and cultivation and found great differences in quantification between cultivation and PCR approaches for *Mycobacterium avium* subsp. *paratuberculosis* (MAP). The authors connected these differences with the incubation time, which is approximately 80 days for MAP. Although dPAB grow relatively fast compared to MAP, long incubation times of at least seven days are required for dPAB quantification. Therefore, the higher quantification levels of dPAB by qPCR compared to quantification on LGA may also be influenced by the slow growth of dPAB as well. With regard to raw milk cheese production, the quantification of higher dPAB levels by qPCR compared to the quantification on LGA is of less concern, as this effect is stronger the more dPAB are present. If the threshold level of 10–30 cfu/mL raw milk is clearly exceeded, minor differences between determined concentrations become irrelevant.

With respect to the needs of the dairy industry, both the quantification of LGA by qPCR present arguments for and against their use. In addition, their strengths and weaknesses have been discussed in detail: while qPCR quantification is more specific and much faster compared to microbiological methods, it presents challenges as the DNA extraction from raw milk, which can critically interfere with the quantification, and requires trained personnel and equipment. In contrast, quantification of dPAB on LGA is time-consuming and comparatively unspecific. Even with an additional identification step after cultivation, only a subset of putative dPAB are identified. Thus, there is a high risk that dPAB levels quantified on LGA will be inadvertently overestimated if accompanying microbiota in raw milk are mistakenly assumed to be dPAB or underestimated if cells enter a VBNC status.

### 3.3. Relationship between dPAB Levels and Farm Management Data

In order to identify which farm management parameters might be affecting the dPAB contamination levels of raw milk, a partition decision tree was constructed for each quantification method (LGA and qPCR—see Supplementary Materials Figure S1). This tree identifies important relationships between dPAB levels in raw milk and farm management/weather data by organizing data according to a relationship between dPAB levels and the response values, which are also listed in the “Column Contributions” in the Supplementary Materials (Figure S1). For both the dPAB quantification on LGA and by qPCR, the milking system, the milk yield produced (kg) per year, referred to as milk output in the tree, the milk type, and the season, were identified as the potentially important response values. Indeed, for both methods, the milking system contributes the most, with a proportion of around 90% (see Supplementary Materials Figure S1 “Column Contributions”), while the remaining proportions are distributed among the factors of season, milk type, and milk yield.

To further assess whether we could identify significant differences in the dPAB contamination levels based on the factors identified using the partition decision, we used mixed model analysis and Tukey’s multiple comparisons. Based on the mixed model analysis, the milking system was identified as an important factor when dPAB levels were quantified by LGA. Significant differences were observed for all four parameters when quantified by qPCR: season, milk type, milk yield, and milking system.

**Figure 4:**
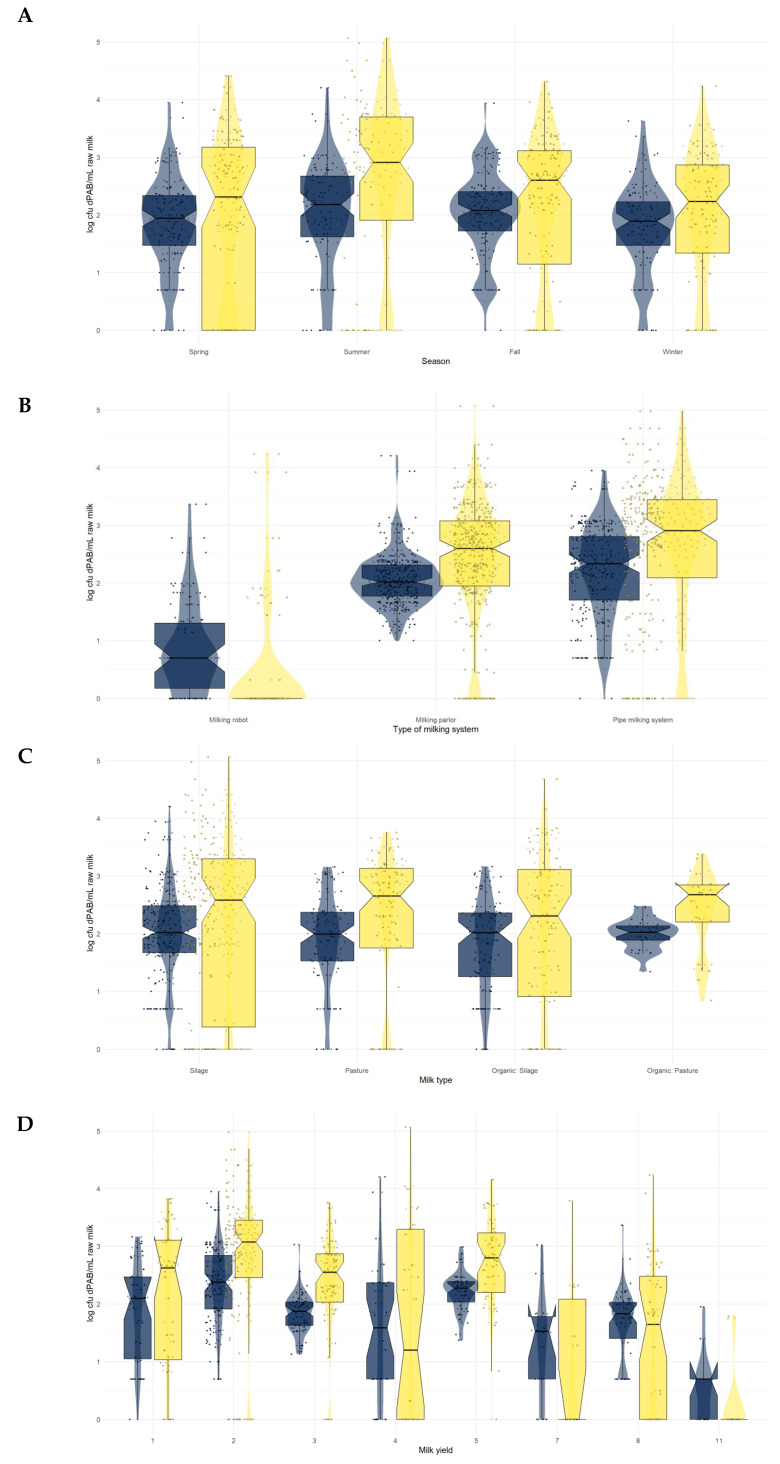
Box plots showing the dPAB levels in raw milk according to the season (**A**), the milking system (**B**), the milk type (**C**), and the milk yield (**D**) quantified on lithium glycerol agar (blue) and by qPCR (yellow). The milk yield is expressed in numbers from 1–11, representing 50,000 kg increments, starting ≤50,000 kg. Box plots represent the median, the first, and the third quartile (25%/75% of the data), and the whiskers extend to the lowest and highest values that are not outliers (max. 1.58 × interquartile range). Each dot represents one sample; dots beyond the whiskers are outliers.

Therefore, these four parameters were included in Figure 4, which presents the dPAB levels in the raw milk samples as cumulative box plots of all farms, including all samples by season (A), milking system (B), milk type (C), and milk yield (D) depending on the quantification method used. Milk yield is indicated by the numbers 1–11, where each number represents a 50,000 kg increment, starting with 1, which corresponds to ≤50,000 kg, and ending with 11, which corresponds to ≥ 500,000 kg. Numbers 6 and 9–11 are omitted because none of the farms had a milk yield in this category. On average, the box plots of the qPCR results (yellow) are stretched wider than the LGA box plots (blue), and the detected dPAB levels by qPCR are higher compared to the results of the quantification on LGA. This is not surprising, considering the mean dPAB level quantified on LGA is 2.0 ± 0.7 log cfu dPAB/mL raw milk, and the mean dPAB level quantified by qPCR is 2.7 ± 0.9 log cfu dPAB/mL raw milk.

As shown in Figure 4A, we have observed seasonal differences in the dPAB levels quantified by qPCR in raw milk (see Figure 2A). Based on Tukey’s multiple comparisons, the levels are significantly higher in summer than in spring (*p* = 0.0006), fall (*p* = 0.0013), and winter (*p* = 0.0016). The partition decision tree did not indicate a strong relationship between dPAB levels and temperature, pasture grazing, or rainfall, respectively. However, it is important to remember that due to the geographical location of Austria, temperatures are much warmer in the summer than in the winter. Propionic acid bacteria are commonly found in the milking system, and a warmer ambient temperature may lead to higher bacterial proliferation rates, thus increasing dPAB contamination levels [4]. Rainfall was also more frequent in summer, with 67% of all milk sampling days experiencing rainfall. The negative effect of rainfall on udder/cow cleanliness has been reported by various authors [35,36,37]. If cattle are out pasture grazing during summer, they will be exposed to rain and consequently have dirtier udders compared to winter, when they remain indoors or have limited outdoor space in a free stall barn. If dPAB levels in raw milk are indeed influenced by teat cleanliness, as is the case for clostridial contamination, the higher dPAB levels in summer may be favored by dirtier udders due to pasture grazing and weather exposure [24]. Furthermore, dPAB have previously been identified on the teat surface, albeit rarely [38]. However, given what is known about clostridial contamination pathways in raw milk, AMS appears to have a negative effect. Anaerobic spore counts in raw milk increased after AMS introduction, which was attributed to inadequate teat cleaning [39]. This would contradict the hypothesis of dPAB transmission via the teat surface, as we observed significantly lower dPAB levels on AMS farms [39]. In a previous study, the most important factor regarding the dPAB contamination of raw milk was the cleanliness of the milking system [4]. All in all, more research is needed to properly evaluate the effects of factors such as season, rain, and udder cleanliness on raw milk dPAB levels. Considering that little is known about dPAB reservoirs outside the dairy environment, this would merit further research.

There is a significant difference in dPAB levels between farms using robotic milking and the other two milking systems, regardless of the quantification method used (see Figure 4B). This was confirmed by Tukey’s multiple comparisons (LGA: AMS vs. milking parlor *p* = 0.0003, AMS vs. pipe milking system *p* = 0.0136; qPCR: AMS vs. both other systems *p* < 0.0001). No matter the quantification method, the lowest dPAB levels were observed on farms using milking robots, and the highest dPAB levels were observed on farms using pipe milking systems. However, the differences between milking parlor and a pipe milking system are less pronounced than between the latter two types of milking systems and milking robots. Considering that many studies report higher bacterial counts/mL of raw milk when automatic milking is used compared to the use of other milking systems, the low dPAB levels in farms with AMS were surprising at first [39,40,41,42]. Even when looking at the mean CC of all milk samples from farms using milking parlors or pipe milking systems (4.2 ± 0.4 log colonies/mL raw milk), the mean CC of milk samples from farms with an AMS (4.2 ± 0.3 log colonies/mL raw milk) is comparable. Although most studies report an increase in CC after transitioning to an AMS, not all do, and there are several parameters that affect the CC in addition to the milking system, such as barn hygiene or equipment sanitation [39,43]. Furthermore, as we did not find any correlations between dPAB levels and the CC, the influence of an AMS on dPAB counts may be limited. A possible explanation for the lower dPAB levels in AMS milk could be cleaning by backflushing or steaming between milkings. Since most dPAB strains are heat sensitive, this could be effective in preventing higher dPAB levels in AMS raw milk [4,43,44]. To the best of our knowledge, we are the first to observe this relationship between dPAB levels in raw milk and the miking system. Due to the potential impact of changes in milk quality resulting from the transition to AMS, producers of PDO cheeses may be cautious about a change in the milking system of their suppliers, even though it has the potential to improve the quality of work and lifestyle of the farmers [39,43]. In terms of dPAB contamination levels, the conversion appears to be beneficial, but further research is needed to confirm this hypothesis.

In contrast, there is less variation when looking at dPAB levels by milk type (see Figure 4C). Especially when quantified on LGA, differences, as in Figure 4B, cannot be observed between dPAB levels according to the milk type. A major difference in the quantification results is the more compact plot shown in the category “organic hay milk”, but this may be due to the comparatively lower number of farms (*n* = 2) included in this study that produce this type of milk. However, dPAB levels in conventional milk were significantly different from dPAB levels in organic milk when quantified by qPCR (*p* = 0.0109). Conventional milk had the highest levels of dPAB contamination, followed by organic hay milk and organic milk, which had the lowest levels of dPAB when quantified on LGA. However, it is important to consider not only the differences in the number of farms supplying the different types of milk but also the differences that may be caused by the milking system used. It remains to be determined whether these differences are really caused by the differences in farm management in the production of the different types of milk, but one factor potentially influencing dPAB levels in raw milk is the feeding of silage dairy farms producing conventional or organic milk, as silage is known to contain (low) levels of dPAB [45,46]. The influence of silage feeding on microbial loads with anaerobic spore formers in raw milk has been demonstrated before, but data on the influence of silage feeding on dPAB levels is limited [47]. A recently published study examined dPAB levels in the forages of 16 silage-free farms, where dPAB levels in all but one feed sample (47 samples in total) were below the limit of detection (2.0 log cfu dPAB/g feed), which is in line with the findings of previous studies on feed contamination [4,38,46]. Most other studies report conflicting results regarding the bacterial load of raw milk from organic and conventional farms but rather report a greater variation in the relative abundance of genera in organic milk or differences in the fatty acid composition of raw milk [48,49]. A difference in the fatty acid composition of milk was also reported by van den Oever et al. [50], who compared the effect of silage and hay feeding. Although they found higher concentrations of essential fatty acids in the milk of cows fed hay, the differences were not significant. However, fatty acid composition is influenced by a number of factors, including feed and breed, so the differences in composition could have a variety of causes, amongst them also the type of milking system [43,49]. AMS are known to cause a decrease in milk fat, which can be problematic for cheese production, especially for traditional cheeses made according to PDO regulations [39,43]. However, in a recent study, Franceschi et al. [43] demonstrated that although the chemical composition and physicochemical properties of milk are affected by AMS, the produced cheeses fully comply with the sensory characteristics according to the PDO regulations. All in all, this shows that milk composition and quality are influenced by a multitude of factors.

The milk yield (see Figure 4D) was the last contributing factor that was identified by the mixed model analysis. When investigating the results of the dPAB quantification levels by qPCR, dPAB levels of farms producing between 200,000–250,000 kg milk/year (category 5-categories see Figure 4D) significantly differed from the dPAB levels of farms producing 350,000–400,000 kg milk/year (category 8, *p* = 0.0020). It is important to note that most of the farms that use a milking robot, which was the type of milking system with the lowest dPAB contamination levels, also have a high annual milk production. Another question that arises with regard to the low dPAB levels found in raw milk from farms using robotic milking is whether this effect is due to the difference in the milking system or because those transitions to a new milking system happened recently. More data are needed to answer this question, as three of the six farms that participated in our study transitioned to robotic milking systems during the screening period. The resulting change in the category “milking system” was taken into account in the statistical analysis. As dPAB are known to be present in biofilms of improperly cleaned milking systems, a recent transition to a new milking system could result in lower dPAB contamination levels [3]. This may not only be because of an absence of pre-existing biofilms in a new system but also because a new system designed according to the latest knowledge on hygienic design may improve the cleaning process compared to the previous milking system. This is essential because a properly set up cleaning regime of the milking system has been identified as one of the crucial parameters to prevent dPAB contamination of raw milk [4].

## 4. Conclusions

In this study, we were able to gain insight into the levels of dPAB contamination and the prevalence of dPAB species in raw milk. Overall, contamination levels ranged from absent in 1 mL of sample material to 4.2 (LGA)/5.1 (qPCR) log cfu dPAB/mL of raw milk. Less than 10% of samples were free of dPAB contamination when examined by microbiological methods, and all known dPAB species were found in raw milk. *P. freudenreichii* was the dominant species in raw milk, followed by *A. jensenii* and *A. thoenii*, while *A. acidipropionici* was rarely found. When analyzed by qPCR, 79.3% of the samples tested positive for dPAB contamination, and contamination with *P. freudenreichii* was more common (96.5%) than contamination with acidipropionibacteria (72.6%). We have shown that dPAB levels differ significantly depending on the season, the milking system used, the size of the farm, and the type of milk produced. In contrast to its often negatively characterized effect on the microbial load of raw milk, milk from farms using AMS had low dPAB contamination levels, which has not been demonstrated before. We quantified higher dPAB levels when farms had a milking parlor, and the highest dPAB levels were detected if a pipe milking system was used. With respect to milk type, we observed the highest dPAB levels in conventional milk while we quantified the lowest levels in organic milk. Furthermore, we have shown that quantification of dPAB on LGA is advantageous compared to YELA. When cultured on LGA, there is a lower proportion of samples overgrown by the non-target microbiota and the growth of *Acidipropionibacterium* spp. appears to be better when using LGA. If a culture-independent approach is preferred, the qPCR assay developed by Turgay et al. [3] is an excellent option, as it reduces pipetting steps and allows the quantification and differentiation of *P. freudenreichii* and the 3dPAB group, even though the detection of critical dPAB levels (≤30 cfu dPAB/mL raw milk) can be difficult. Considering the dairy industry’s needs, both methods are well-suited, albeit each one has its drawbacks and limitations: cultivation of dPAB is time-consuming and non-selective; qPCR is laborious and comparatively expensive. A method that combines speed, selectivity, and simplicity for the operator is still lacking. In conclusion, the detection of (low-level) dPAB contamination remains a challenge today, regardless of the quantification method used.

## Data Availability

The original contributions presented in the study are included in the article/Supplementary Materials, further inquiries can be directed to the corresponding author.

## Supplementary Materials

The following supporting information can be downloaded at https://www.mdpi.com/article/10.3390/foods13121921/s1, Figure S1: Partition decision tree and corresponding column contributions for both quantification methods (LGA/qPCR).
