# Supplementary material for: One-Year Monitoring of Prevalence and Diversity of Dairy Propionic Acid Bacteria in Raw Milk by Means of Culture-Dependent and Culture-Independent Methods
Source: Foods. 2024 Jun 18;13(12):1921. doi: 10.3390/foods13121921 (PMC11203294; doi:10.3390/foods13121921)
Supplement: Supplementary file 1 [file foods-13-01921-s001.zip › foods-3046366-supplementary.pdf]

Supplementary Figure S1: Partition decision tree and corresponding column contributions for both quantification methods (LGA/qPCR).

Lithium glycerol agar: decision tree and column contributions

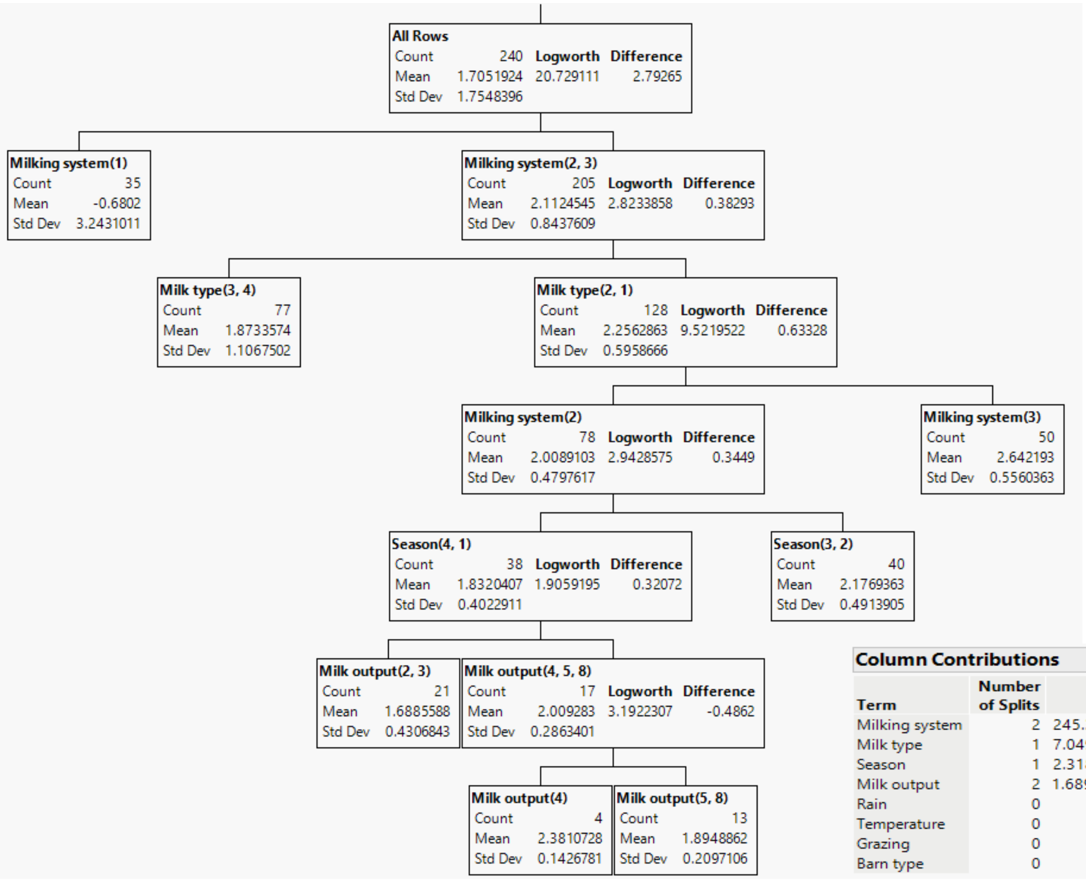

Column Contributions

Term	Number of Splits	SS	Portion
Milking system	2	245.374625	0.9569
Milk type	1	7.04990331	0.0275
Season	1	2.31805808	0.0090
Milk output	2	1.68941701	0.0066
Rain	0	0	0.0000
Temperature	0	0	0.0000
Grazing	0	0	0.0000
Barn type	0	0	0.0000

qPCR: decision tree and column contributions

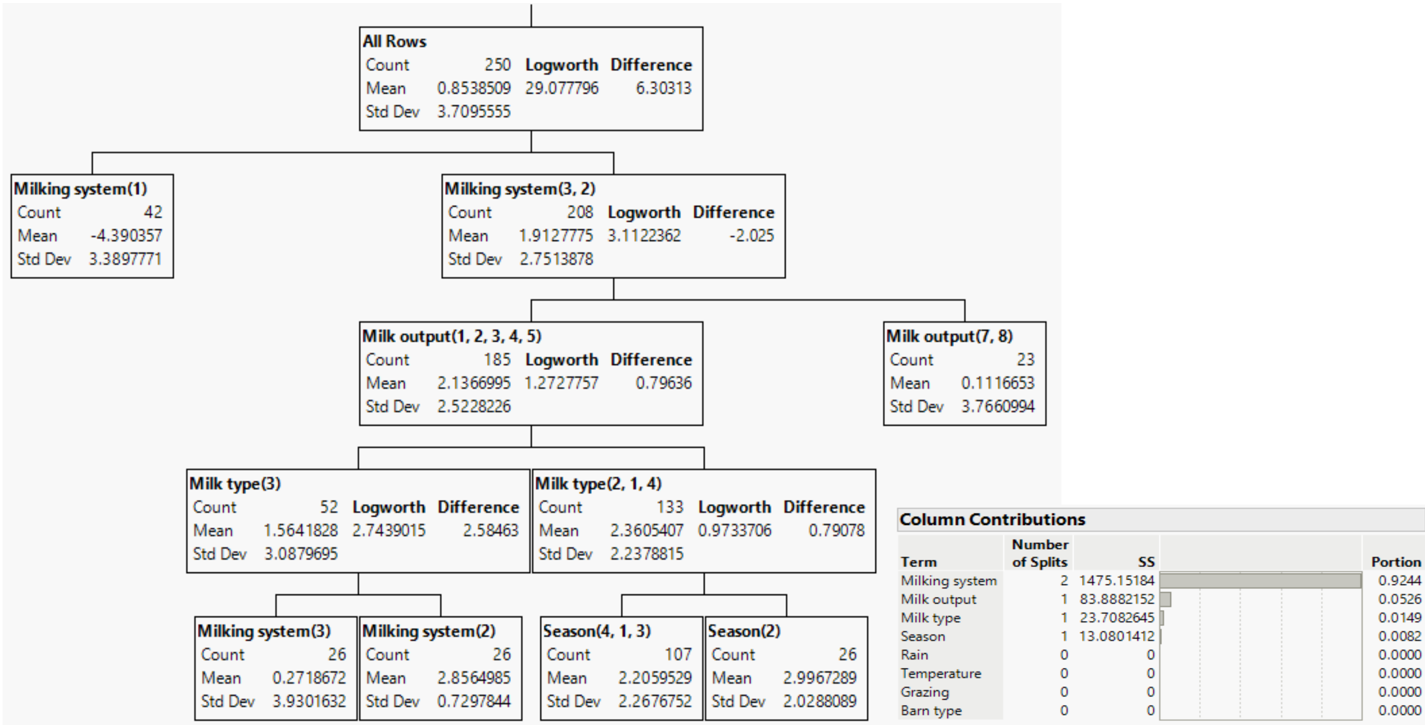

Column Contributions

Term	Number of Splits	SS	Portion
Milking system	2	1475.15184	0.9244
Milk output	1	83.8882152	0.0526
Milk type	1	23.7082645	0.0149
Season	1	13.0801412	0.0082
Rain	0	0	0.0000
Temperature	0	0	0.0000
Grazing	0	0	0.0000
Barn type	0	0	0.0000
